# Virtual Patients in Clinical Trials for Drug Development: A Narrative Review

**DOI:** 10.7759/cureus.85380

**Published:** 2025-06-04

**Authors:** Jan A Kleeberger

**Affiliations:** 1 Department of Cardiology, Universität Zürich, Zürich, CHE; 2 Center for Translational and Experimental Cardiology (CTEC), Universität Zürich, Zürich, CHE

**Keywords:** biosimulation, clinical trials, drug development, machine learning, pharmaceutical industry, statistical methods, virtual patients

## Abstract

The pharmaceutical industry faces significant challenges due to the prolonged development timelines, high failure rates of innovative drugs, and escalating regulatory demands for robust data. A novel solution to address these challenges is the utilization of virtual patient cohorts to simulate drug effects in computer models. This review article provides an overview of the application of virtual patient cohorts in drug development and the diverse methodologies employed, based on a comprehensive literature review. Complementing previous reviews, this work emphasizes the practical applications of virtual patients across various disease conditions, especially rare diseases where patient recruitment is particularly challenging. It also highlights the challenges of conventional trial methodologies, such as limited generalizability, ethical concerns, and recruitment barriers, and discusses virtual patients as a potential solution to these longstanding issues. Since all literature was included according to the judgment of a single reviewer, a potential selection bias must be taken into account when reading this article. Virtual patients can be generated as digital twins through statistical inference or by randomly assigning parameters, followed by plausibility assessments, with the method choice depending upon study objectives and available data. These cohorts find utility across all clinical phases of drug development. The principal advantages of leveraging virtual patient cohorts include potential cost savings through heightened development success and increased innovation. Moreover, they offer improved representation of patient groups often marginalized in drug development efforts. However, it is imperative to acknowledge the computational nature of virtual patients, which can yield erroneous outcomes and necessitate substantial expertise and computational resources. Currently, there is a lack of standardized protocols for generating and utilizing virtual patient cohorts. Nonetheless, virtual patient cohorts hold promise in fundamentally transforming drug development and patient treatment approaches. By creating realistic virtual patients, more efficient and personalized drug development strategies can be pursued. Integrating this technology alongside in vitro and in vivo studies, while considering their respective limitations, might significantly enhance the success rate of drug development across the pipeline, ultimately advancing patient health outcomes.

## Introduction and background

The development of new pharmaceuticals is a complex and costly endeavor, often characterized by high failure rates and lengthy timelines. The traditional dogma of drug development, including in vitro experiments, animal testing, and clinical trials, has proven unsatisfying to meet the growing demand for innovative and effective treatments. This is reflected in a low success rate of about 10% of active agents, which makes it from patent protection to market access [1]. In recent years, the concept of virtual patients has emerged as a promising solution to these challenges [2]. Virtual patients are computer-generated simulations that mimic the clinical characteristics of real patients, offering a novel approach to drug development and enabling researchers to simulate clinical trials without involving human participants. For instance, a virtual patient cohort with type 2 diabetes can be created by analyzing real-world clinical data, such as glucose levels, weight, and medication history. These virtual patients can then be used to simulate how different dosages of a new drug might impact blood sugar control, reducing the need for early-phase human trials while providing valuable insights into drug efficacy. This article provides an overview of virtual patients in drug development for various conditions, especially rare diseases. In addition, a generalizable framework for the creation of virtual patients should be developed and illustrated by a schematic to obtain a simplified overview to be easily grasped by clinician scientists. Finally, this article identifies specific issues and offers a balanced analysis of the state of the field and its future directions based on the existing literature, especially considering recent developments in the field of artificial intelligence (AI)/machine learning (ML), with a critical appraisal.

Phases of drug development

Drug development typically involves several phases, beginning with preclinical research and progressing through multiple stages of clinical trials. Preclinical research includes in silico experiments, cell culture studies, and animal testing to evaluate the pharmacological and toxicological properties of new compounds [3]. Clinical trials are divided into four phases: Phase I involves first-in-human studies with healthy volunteers to assess safety and determine appropriate dosage. Phase II consists of pilot studies in patients to evaluate the treatment’s efficacy and identify potential side effects. Phase III includes large-scale trials in patient populations to confirm effectiveness and monitor adverse reactions. Finally, Phase IV takes place after the treatment is on the market and focuses on gathering additional information about long-term risks, benefits, and optimal use.

Challenges in drug development

The drug development process is fraught with challenges, including high costs, stringent regulatory requirements, and technical uncertainties [4]. Only about 10% of drug candidates successfully transition from patenting to market approval [1]. The average time from patenting to FDA approval is approximately 10 years, with costs exceeding $2.87 billion per new drug [5]. Consequently, there is a big potential to save costs. Clinical trials also involve risks to participants and often require extensive animal testing, leading to ethical concerns. Since few patients are available for recruitment, rare diseases and specific subpopulations of more common conditions pose significant challenges for patient recruitment. These challenges underscore the need for innovative methods to improve the efficiency and success rates of drug development. Virtual patients may address these challenges by enabling simulated trials, which can be more efficient, scalable, and inclusive.

For instance, in rare diseases like cystic fibrosis, where patient recruitment is limited, virtual cohorts can facilitate robust clinical studies that would otherwise be impractical [6].

## Review

Methods

Literature Search

**Figure 1:**
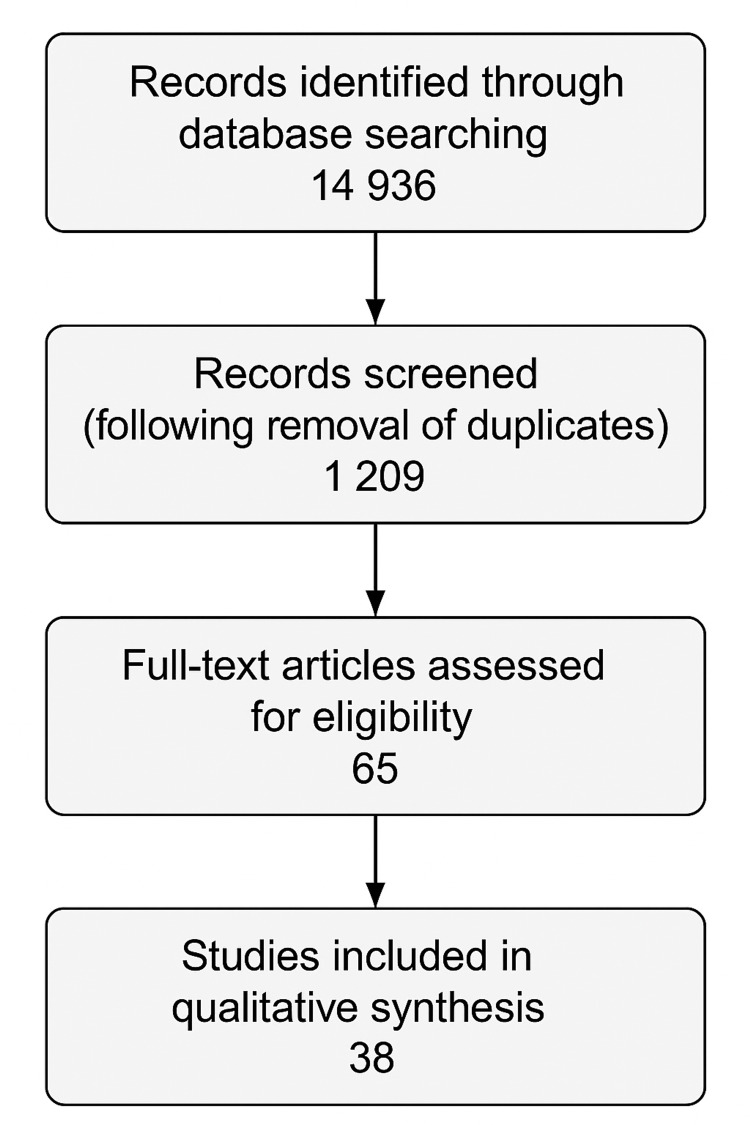
Literature search flowchart

A comprehensive literature search was conducted using databases such as MEDLINE via PubMed and Google Scholar. The search covered publications from 1950 to March 2025, focusing on both German and English language sources. A single reviewer screened all search results for the whole review; no automation tools were used. The search terms included “virtual patients drug development”, “virtual populations in drug development”, “machine learning clinical trial drug development”, and “digital twin drug development”. No Boolean operators were employed. This yielded 14,936 results in total, and 38 references were used for the creation of this review. Figure 1 depicts the screening workflow. The risk of bias within the trials included in this review was not systematically assessed.

Inclusion and Exclusion Criteria

The inclusion criteria for the review were studies that discussed the creation, application, and evaluation of virtual patients in drug development, according to the authors’ judgment. Since this is a subjective selection, a selection bias must be considered when reading this review. Exclusion criteria included studies that did not provide sufficient detail on the methodologies used or that focused solely on preclinical drug discovery without addressing clinical trial simulations. In addition, articles that were about topics other than virtual patients and clinical trial simulations were excluded. An example that occurred frequently entails literature about virtual clinical trials in the sense of remote clinical trials leveraging modern communication technology to avoid in-person follow-up visits.

Data Extraction and Analysis

Data were extracted from the selected studies, including information on the methodologies used to create virtual patients, the applications of these models in drug development, and the outcomes of virtual clinical trials. The extracted data were analyzed to identify common themes, advantages, and limitations of virtual patient models.

Virtual patients: concept and methodologies

Definition and Importance

Virtual patients are computer-generated models that simulate the clinical characteristics of real patients [7]. These models are used within in silico studies to predict the effects of drugs without the need for initial human or animal testing. Virtual patient cohorts, or groups of virtual patients, are central to these studies, allowing researchers to theoretically conduct trials entirely within a computer environment [8].

Methodologies for Creating Virtual Patients

Several methodologies are employed to create virtual patients, each with its own advantages and limitations. Table 1 gives an overview of the methods discussed in this review.

**Table 1:** Overview of methods for generating virtual patients This table discusses the advantages and disadvantages of various approaches used to generate virtual patients. ABM, agent-based modeling; AI, artificial intelligence; ML, machine learning

Method	Advantages	Disadvantages
ABM	Models individual patient interactions; useful for studying complex behaviors and outcomes like disease transmission and immune responses; applied in oncology for predicting treatment efficacy	Requires significant computational resources; limited scalability for very large populations
AI and ML	Analyzes large datasets for patterns and predictions; enhances simulation accuracy; facilitates the creation of synthetic datasets for rare diseases and small samples	High computational demand; susceptible to the “black box” problem, reducing trust and interpretability; risks of bias in training data
Digital twins	Real-time simulations and updates based on clinical data; enables high temporal resolution and real-time effects of interventions	High dependency on high-quality, real-time data; expensive and computationally intensive to maintain
Biosimulation/statistical methods	Uses established mathematical and statistical models (e.g., Monte Carlo simulations, regression analysis); predicts diverse clinical scenarios and outcomes; cost-effective for small-scale data modeling	Limited by the assumptions and accuracy of the models; may oversimplify complex systems, leading to reduced generalizability

Agent-based modeling (ABM): ABM simulates the interactions of individual agents (virtual patients) within a system, allowing for the study of complex behaviors and outcomes. This method is particularly useful for modeling disease transmission and immune responses [3,9,10]. For example, ABM has been applied in oncology to simulate tumor progression and the effects of combination therapies, enabling researchers to predict treatment efficacy under various scenarios [10].

AI and ML: AI and ML techniques analyze large datasets to identify patterns and make predictions based on probabilities. These methods are used to create virtual patients by learning from real patient data. ML can enhance the accuracy of simulations and uncover hidden relationships within the data [10-12]. Since the release of the chatbot ChatGPT (OpenAI, San Francisco, USA), AI has become a prominent topic, demonstrating its versatility in automating complex processes and facilitating innovative solutions across industries, including healthcare. Within the context of drug development, AI/ML is particularly useful for generating synthetic datasets to augment small sample sizes in clinical trials [13] and for predicting outcomes in rare diseases [14], where traditional trial designs face significant recruitment challenges.

Digital twins: Digital twins are virtual replicas of real patients, created using data from actual clinical records [15]. These models are continuously updated with new data, allowing for real-time simulations and predictions [16-18]. Since the temporal resolution is high, this method can also be used to simulate real-time effects of different therapeutic interventions [19]. A recent example is the visualization of myocardial scar tissue by using a digital twin created from imaging data. This dataset can be further used to test interventions, such as targeted ablation procedures [20].

Biosimulation/statistical methods: Biosimulation uses mathematical models to simulate biological processes [21]. This method can be applied to both small and large scales of data, depending on the complexity of the system being studied [22]. Techniques such as ordinary differential equations (ODEs) and Monte Carlo simulations are commonly used in biosimulation [23]. Other typically used statistical methods, including regression analysis and bootstrapping, are used to generate virtual patient data by extrapolating from existing datasets [24]. These methods can create realistic patient cohorts that reflect the diversity of real populations. Regression analysis is a method that can be used to predict the relationship between drug dosage and patient response [25]. For example, a regression model may be used to analyze historical clinical trial data to predict how different dosages of a new drug will affect blood pressure in virtual patients [26]. Bootstrapping is a technique that involves repeatedly resampling a dataset to create multiple simulated samples. For instance, bootstrapping can be used to estimate the variability in patient responses to a drug by generating numerous virtual cohorts from a smaller real-world dataset [24]. Monte Carlo simulations, on the other hand, are simulations that use random sampling to model complex systems and assess the impact of uncertainty. In drug development, Monte Carlo simulations can predict the probability of different clinical outcomes based on various treatment scenarios [27-28]. Biosimulation and statistical methods are particularly valuable in optimizing clinical trial design and drug dosage. They can help in identifying ideal dosages that balance efficacy and safety, modeling disease progression to determine the most appropriate intervention points, and predicting the success rate of clinical trials before they are initiated. For example, biosimulation has been extensively used in pharmacokinetics and pharmacodynamics modeling, where it enables researchers to simulate drug absorption, distribution, metabolism, and excretion to predict patient-specific outcomes. These techniques are also critical in rare disease research, where limited patient populations make traditional recruitment and trial designs impractical [29].

General Approaches to Generating Virtual Patient Cohorts

There is no universally accepted method for generating virtual patient cohorts [8]. Generally, three approaches are used to generate parameters for all patients within a virtual cohort: (1) creating virtual patients as digital twins, which involves generating virtual patients based on specific real individuals; (2) artificially generating patient characteristics by applying statistical inference from existing datasets, such as resampling techniques or ODEs; and (3) randomly generating parameters, where parameters are randomly sampled and then checked for physiological plausibility - an example of this is Monte Carlo simulation.

Creating a virtual patient cohort requires expertise across several domains. Professionals must have a strong foundation in pharmacology and medicine to understand drug mechanisms, disease pathophysiology, and clinical trial design. Mathematical and statistical modeling skills, including techniques such as regression, Bayesian inference, and differential equations, are essential for building robust frameworks [2]. Computational science expertise is needed for programming, ML, and simulation algorithm development. Data scientists and biostatisticians play a key role in managing large datasets and ensuring the diversity of patient representation. Familiarity with biosimulation platforms is also important, along with knowledge of regulatory requirements to ensure compliance and acceptance of the models [30].

Selection of the Appropriate Approach

To select the most suitable approach, it is first necessary to precisely formulate the research question that the virtual patient cohort is intended to answer [31]. Researchers can also refer to comparable in vivo studies conducted for similar drugs to create the design for the virtual trial. The main factors for choosing the appropriate model are the complexity required to address the research question and the available time and resources for model creation. Additionally, researchers must consider the broadness of the existing data pool. The easiest approach is statistical inference using regression analyses of a few variables and parameters, while the most complex models may involve ML-based models of molecular pathways with high detail resolution [32].

Model Creation and Parameter Setting

Once a mathematical foundation for model creation is selected, the corresponding model is created on the computer. The parameters of the model are then set either based on the data foundation, values from the literature, or extrapolation from other populations.

Sensitivity Analysis and Model Identifiability

Regardless of the chosen method, a sensitivity analysis must be conducted, and the model’s identifiability must be verified [8]. A sensitivity analysis examines how changes in input parameters affect the model’s response. This helps determine which parameters should remain constant across all virtual patients and which should vary individually, defining the unique characteristics of each virtual patient. Model identifiability refers to the ability of the model to derive parameter values from comparisons with previous observations. If the data are limited or incomplete, certain parameter values may not be well defined, leading to a broad spectrum of acceptable values that qualitatively match the reference data. Performance can be improved by identifying these parameters through the identifiability analysis and estimating their values from other sources or studies.

Generation and Grouping of Virtual Patients

The actual generation of virtual patients and their grouping into cohorts follows. The three methods mentioned above can be employed. Rieger et al. published an approach involving the random generation of parameters using a form of Monte Carlo simulation, followed by mathematical verification of the physiological plausibility of the resulting parameters and their alignment with existing clinical data [33]. These two steps can be repeated as needed until the cohort of plausible virtual patients reaches the desired size. A selection step may be necessary if only a specific subpopulation is to be considered, such as virtual patients over 80 years of age.

Simulation and Analysis

Subsequently, simulations and analyses specific to the research question can be conducted with the virtual patients in the context of an in silico study. Virtual patients can also be used as synthetic control arms to complement in vivo studies [29]. Throughout the process, the model’s results should be continuously scrutinized for plausibility, and the success of the endeavor largely depends on the expertise of the data specialists involved.

Quality Control

**Figure 2:**
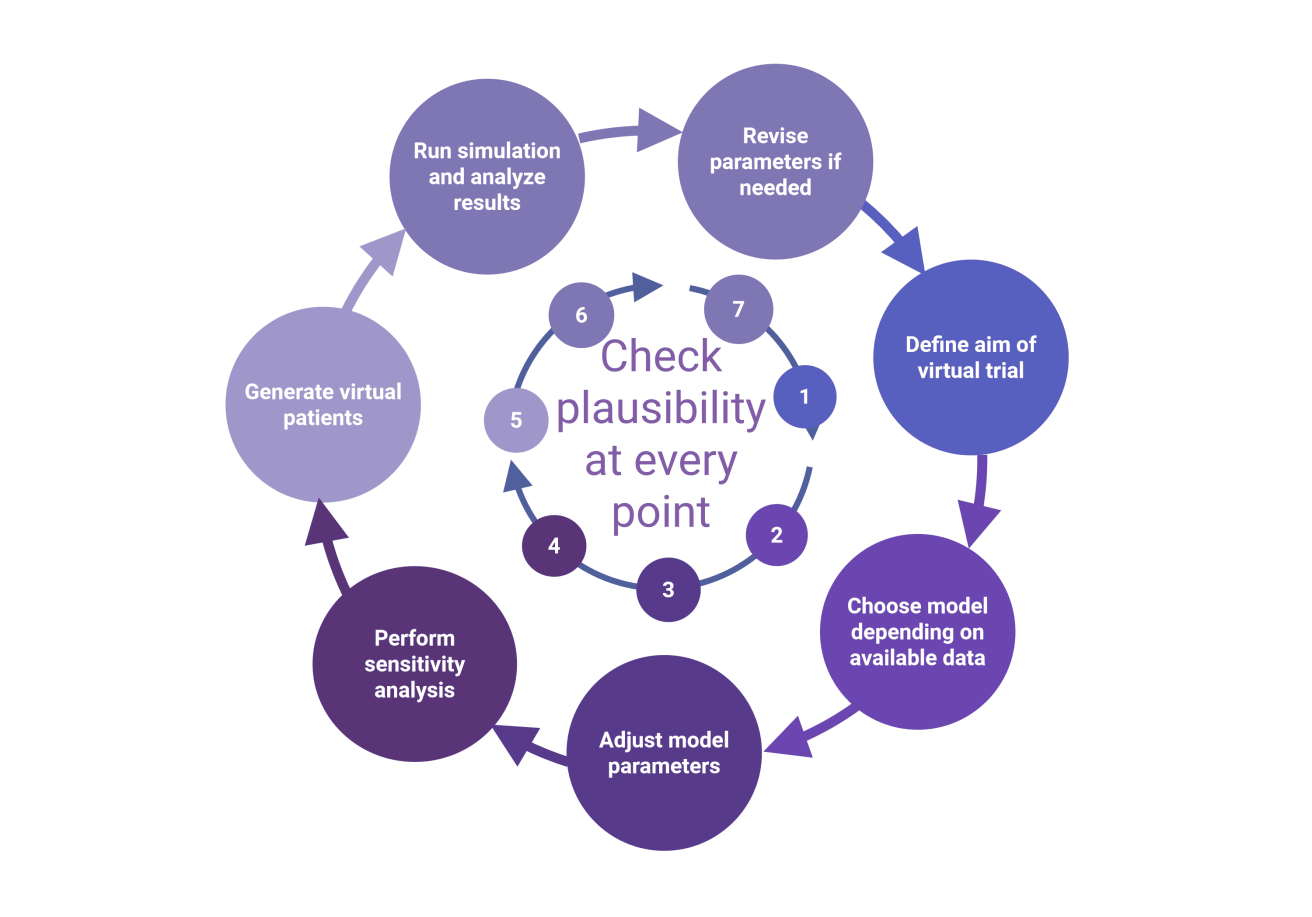
Cycle for generation of virtual patients

At the end of the process, a quality control of the results is conducted to ensure their internal and external validity, particularly by comparing them with the original data. Various sources of error may arise, such as inadequately formulated research questions or mismatches with available reference data. The model itself or the derived parameters may also be incorrect. Implausible results may indicate previously unknown behaviors of the underlying biological processes, necessitating further in vivo experiments. Figure 2 illustrates the process of generating a virtual patient cohort. As shown, the process is cyclical and can be repeated multiple times, not necessarily ending with the receipt and quality control of the results. Validation of virtual patient models ensures that they reliably represent the target population and are suitable for their intended use. This involves internal validation, where model outputs are compared to real-world data from the same dataset, and external validation, which tests the model against independent datasets to evaluate generalizability. Predictive validation assesses the model’s ability to forecast real-world outcomes, such as drug efficacy or safety. Finally, the validation process must align with regulatory frameworks, such as the FDA’s guidelines for model-informed drug development, to establish credibility and compliance [30,34].

Applications and benefits

Enhancing Drug Development

Virtual patients offer several benefits in drug development. They can lead to cost reduction and more time efficiency along the drug development pipeline:

Cost reduction: By simulating clinical trials, virtual patients can significantly reduce the costs associated with traditional drug development [35]. This effect is not only caused by a higher efficiency, but also by a lower number of non-virtual patients required to reach the statistical power necessary for valid results.

Time efficiency: Virtual trials can be conducted more quickly than in vivo studies, accelerating the development timeline [10].

Improved success rates: Virtual patients can help identify promising drug candidates earlier in the development process, reducing the likelihood of late-stage failures [36].

Ethical considerations: Virtual trials reduce the need for animal testing and the risks to human participants.

Addressing Rare Diseases

Virtual patients are particularly valuable in the study of rare diseases, where patient recruitment for clinical trials is challenging because of the scarcity of potential trial participants. By creating large cohorts of virtual patients, researchers can conduct more robust studies and develop treatments for conditions that might otherwise be neglected.

Challenges and limitations

Despite their potential, virtual patients also present several challenges. These challenges arise mainly from the nature of virtual patients being a computer simulation:

Model Accuracy

The accuracy of virtual patient models depends on the quality of the underlying data and the assumptions made during model creation. Incorrect assumptions can lead to misleading results [7].

Computational Resources

Creating and simulating virtual patients requires significant computational power and expertise.

Regulatory Acceptance

Regulatory bodies like the FDA and European Medicines Agency are still developing guidelines for the use of virtual patients in drug development. Ensuring that virtual trials meet regulatory standards is crucial for their widespread adoption.

Data Privacy

The use of real patient data to create virtual patients raises concerns about data privacy and security.

Black Box Problem of AI

AI/ML models, particularly complex ones like deep learning networks, operate using intricate mathematical transformations that are often incomprehensible to human users. This issue was coined the “black box” problem in AI/ML and refers to the lack of transparency in how these systems arrive at their conclusions or predictions [37]. This opacity becomes particularly problematic when applying AI/ML to create virtual cohorts for drug development. If the decision-making process of a model is not well understood, it can lead to a lack of trust among regulators, clinicians, and other stakeholders. For example, biases in the training data - such as overrepresentation of certain demographics - may propagate into the virtual cohort, potentially skewing results. Furthermore, without transparency, it is challenging to identify errors or unintended consequences in model outputs, such as misrepresenting disease progression or drug response. These issues raise ethical concerns, particularly in scenarios where treatment decisions or regulatory approvals may depend on the outcomes of such opaque models [34].

Future directions

The field of virtual patients in drug development is rapidly evolving. To reduce the challenges described above, future research should mainly focus on the following:

Standardization

Developing standardized methodologies for creating and validating virtual patients to ensure consistency and reliability.

Integration With Traditional Methods

Combining virtual trials with traditional in vivo and in vitro studies to leverage the strengths of virtual and real-world approaches.

Regulatory Frameworks

Establishing clear regulatory guidelines for the use of virtual patients in drug development.

Advancements in AI and ML

Enhancing AI and ML techniques to improve the accuracy and predictive power of virtual patient models, while being aware of the disadvantages inherent in these tools. Working toward this goal, it is of paramount importance to address the “black box” problem. This requires techniques like explainable AI, which aims to provide interpretable insights into model behavior, ensuring the credibility and safety of AI-generated virtual cohorts [38]. In addition, high-quality training data is required to increase overall model accuracy and the generalizability of the data.

## Conclusions

Virtual patients represent a transformative approach to drug development, offering significant benefits in terms of cost, time, and ethical considerations. While considerable challenges remain, ongoing research and advancements in technology are likely to overcome these obstacles, paving the way for more efficient and successful drug development processes. As the field continues to evolve, virtual patients have the potential to revolutionize the pharmaceutical industry and improve patient outcomes worldwide.

## Appendices

**Table 2:** Overview of the literature used in the development of this narrative review ABM, agent-based modeling; AI, artificial intelligence; ML, machine learning; ODE, ordinary differential equation; XAI, explainable AI

Reference (first author)	Key focus	Type of study
van der Graaf	Probability of success in drug development	Journal article focused on clinical pharmacology and drug success rates
Viceconti	In silico clinical trials and their transformative potential in biomedical industries	Review article discussing virtual patient applications in drug development
Gassmann	Leading pharmaceutical innovation strategies	Book addressing innovation processes in the pharmaceutical industry
Hay	Clinical development success rates for investigational drugs	Research article analyzing clinical trial success rates
DiMasi	Innovation in pharmaceutical R&D and cost estimates	Journal article estimating costs and timelines in drug development
Carlier	Virtual trials for pediatric orphan diseases	Study showcasing in silico trials in rare diseases
Pappalardo	Overview of in silico clinical trials and early applications	Journal article introducing concepts and applications of virtual patients
Surendran	Generating virtual patient cohorts with applications in oncology	Book chapter exploring methodologies for creating virtual patient cohorts
Andrae	Agent-based modeling techniques	Book on agent-based modeling approaches
Pin	Using agent-based modeling in early drug development	Journal article presenting examples of ABM in pharmacology
Fakhouri	AI and ML applications in drug development.	FDA white paper on artificial intelligence in drug development
Gates	ML efficiencies in clinical trial data extraction	Research article evaluating AI tools in trial data extraction
Nikolopoulos	Generative AI in augmenting clinical trial sample sizes	Research study on AI algorithms for synthetic datasets
Irissarry	AI applications in orphan drug research	Research article exploring AI use in rare disease drug development
Armeni	Digital twins in healthcare	Review article examining the applications of digital twins in medicine
Grieves	Definitions, characteristics, and use cases of digital twins	Series of articles/book chapters providing foundational knowledge on digital twins
Manetti	Application of digital twins in myocardial intervention simulations	Study on using digital twins for simulating medical interventions
Waight	Personalized heart digital twins for ventricular tachycardia	Case study applying digital twins in cardiology
Niklas	Biosimulation and statistical methods in drug development	Articles describing mathematical models like ODE and Monte Carlo simulations
Niederer	Multivariate and discrete resampling techniques for virtual patient creation	Journal article discussing statistical resampling for virtual cohorts
Garbade	Quantitative retrospective modeling for rare diseases	Research on regression analysis for virtual patient modeling in rare diseases
Schneider	Applications of linear regression in clinical trial predictions	Overview of linear regression methodologies
Muraro	Monte Carlo simulations for medical and pandemic research	Studies applying Monte Carlo methods in diverse healthcare contexts
Thorlund	Synthetic and external controls in clinical trials	Using virtual cohorts as control arms in trials
FDA	FDA guidelines for AI and ML in drug development	FDA discussion papers on regulatory frameworks
Craig	Practical guide for model-based virtual clinical trials	Research article on best practices in virtual trial design
Wang	Future perspectives on virtual patients in medicine	Review article discussing emerging trends and innovations
Rieger	Improved methods for generating virtual populations	Study introducing advanced techniques for creating robust virtual cohorts
Castelvecchi	Challenges of AI “black box” problem	Discussion article on ethical and interpretative issues in AI applications
Tjoa	XAI for medical applications	Research on XAI approaches to enhance trust in AI-generated models
Jentzsch	Costs and causes of oncology drug attrition	Research on financial implications of drug failures
Subbiah	Evidence-based medicine advancements using virtual patients	Article discussing the integration of virtual trials into traditional medical practices
